# Changes in Optimal Childcare Practices in Kenya: Insights from the 2003, 2008-9 and 2014 Demographic and Health Surveys

**DOI:** 10.1371/journal.pone.0161221

**Published:** 2016-08-17

**Authors:** Dennis Juma Matanda, Helga Bjørnøy Urke, Maurice B. Mittelmark

**Affiliations:** 1 Population Council, Nairobi, Kenya; 2 Multicultural Venues in Health, Gender and Social Justice Research Group, University of Bergen, Bergen, Norway; 3 Department of Health Promotion and Development, Faculty of Psychology, University of Bergen, Bergen, Norway; TNO, NETHERLANDS

## Abstract

**Objective(s):**

Using nationally representative surveys conducted in Kenya, this study examined optimal health promoting childcare practices in 2003, 2008–9 and 2014. This was undertaken in the context of continuous child health promotion activities conducted by government and non-government organizations throughout Kenya. It was the aim of such activities to increase the prevalence of health promoting childcare practices; to what extent have there been changes in optimal childcare practices in Kenya during the 11-year period under study?

**Methods:**

Cross-sectional data were obtained from the Kenya Demographic and Health Surveys conducted in 2003, 2008–9 and 2014. Women 15–49 years old with children 0–59 months were interviewed about a range of childcare practices. Logistic regression analysis was used to examine changes in, and correlates of, optimal childcare practices using the 2003, 2008–9 and 2014 data. Samples of 5949, 6079 and 20964 women interviewed in 2003, 2008–9 and 2014 respectively were used in the analysis.

**Results:**

Between 2003 and 2014, there were increases in all health facility-based childcare practices with major increases observed in seeking medical treatment for diarrhoea and complete child vaccination. Mixed results were observed in home-based care where increases were noted in the use of insecticide treated bed nets, sanitary stool disposal and use of oral rehydration solutions, while decreases were observed in the prevalence of urging more fluid/food during diarrhoea and consumption of a minimum acceptable diet. Logit models showed that area of residence (region), household wealth, maternal education, parity, mother's age, child’s age and pregnancy history were significant determinants of optimal childcare practices across the three surveys.

**Conclusions:**

The study observed variation in the uptake of the recommended optimal childcare practices in Kenya. National, regional and local child health promotion activities, coupled with changes in society and in living conditions between 2003 and 2014, could have influenced uptake of certain recommended childcare practices in Kenya. Decreases in the prevalence of children who were offered same/more fluid/food when they had diarrhea and children who consumed the minimum acceptable diet is alarming and perhaps a red flag to stakeholders who may have focused more on health facility-based care at the expense of home-based care. Concerted efforts are needed to address the consistent inequities in the uptake of the recommended childcare practices. Such efforts should be cognizant of the underlying factors that affect childcare in Kenya, herein defined as region, household wealth, maternal education, parity, mother's age, child’s age and pregnancy history.

## Introduction

The past decades have seen intensive global efforts in combating child mortality and morbidity, and promoting child health and wellbeing. The Millennium Development Goals have been an important global initiative, inspiring a range of health promotion initiatives. Although substantial progress has been made, the 2015 Millennium Development Goals report shows that the challenge of ensuring progress in child health across the globe persists [1]. In 2012, it was reported that six million children died annually before reaching five years; half of which lived in sub-Saharan Africa [1]. Most of the under-five deaths are due to preventable diseases, like pneumonia, diarrhea, and malaria [1]. In Kenya, the 2014 Demographic and Health Survey indicates that one in every 19 children die before they attain the age of five years [2]. The survey also shows that a quarter of Kenyan children under the age five are stunted. Poor child health has many carryon effects manifest in poor adult health, low educational attainment, and facilitation of intergenerational poverty [3].

Society’s medicalized orientation leads many child health experts, and perhaps many parents, to focus mostly on disease diagnosis, risk factor prevention and treatment. Yet the fundamental factor in promoting good child health is the quality of ordinary childcare in the home [4]. A neglected or mistreated child cannot thrive no matter how sophisticated the health and medical services may be. Conversely, a nurturing home environment may foster child health even under harsh living conditions including poor availability of health care services. The importance of good quality childcare in promoting child and subsequent adult health has been firmly conceptualized in the child health and development field [5–7]. Empirically, studies have shown the enormous potential of care in scaling down mortality rates [8,9].

In the past decades, a plethora of disease prevention and health promotion campaigns and programmes have been implemented to combat child malnutrition, malaria, HIV/AIDS, diarrhea, and promote healthy and happy children. In this work, the role of childcare practices has been given increased attention. The United Nations Children’s Fund (UNICEF) in collaboration with the World Health Organization (WHO) identified 12 family and community practices essential in enhancing child survival, growth and development [10]. Among these practices are initiating care before birth (with the pregnant woman attending antenatal care), providing full course immunization before the child’s first birthday, disposing properly of the child’s feces, placing the sleeping child under an insecticide treated bed net (ITN), engaging in nutritious complementary feeding at the child’s weaning, caring for the sick child by offering more to eat and drink including breastfeeding, and ensuring that the child with an infection gets treatment [10].

Despite the fundamental importance to child health of basic childcare in the home, worrying gaps exist between the ideal and reality [11]. Notwithstanding the increase in the number of pregnant mothers attending at least one ANC visit, few women in the south of the Sahara attend the recommended four or more visits [12,13]. Immunization rates have improved but one-in-five children are unimmunized [10]. Despite a sustained campaign urging the use of ITNs to prevent malaria, ITNs are still underutilized [14]. Home care to reduce diarrhea is effective, but key practices are not yet fully embraced [15–17].

In Kenya a range of national strategies, policy guidelines and programmes (referred to hereafter as ‘health promotion activities’ or ‘campaigns’) have been implemented with the aim of improving the health and wellbeing of the population as a whole and of children in particular. Some of the notable programmes that were underway in the period between 2003 and 2014 are:

- the Kenya Economic Recovery Strategy for Wealth and Employment Creation 2003–2007 [18],
- the National Health Sector Strategic Plan I 1999–2004 [19],
- the National Health Sector Strategic Plan II 2005–2010 [20],
- the National Expanded Programme on Immunization [21],
- the National Malaria Strategy 2001–2010 [22],
- the National Strategy on Infant and Young Child Feeding 2007–2010 [23],
- the Integrated Management of Childhood Illness Strategy [24].
- the 2004, 2007 and 2013 changes in policy on user fees in health facilities in Kenya [25,26].

It is reasonable to expect that the sum of these extensive efforts would have contributed to improved childcare in Kenya, and that the prevalence of health promoting childcare practices would have increased significantly over the 11-year period under study. While cause and effect relationships between health promotion activities and health practices changes cannot be established with certainty, it can at least be expected that during the course of multiple, intensive, long-running activities, some gains in childcare would be manifest. The only practical way to assess progress is to study patterns of optimal childcare practices across time, in populations as a whole and in various sociodemographic sub-groups.

The role of sociodemographic and -economic factors is widely studied in relation to child health and nutrition in developing countries [27–31]. Research with a particular focus on urban-rural differences has found that children tend to fare better in urban compared to rural areas [27–31]. However, several of the same studies have also examined the role of socioeconomic and other social factors in the urban-rural differences, and concluded that a large proportion of the differences is due to differences in socioeconomic status [27,28,30]. A review of several countries of sub-Saharan Africa found that in Kenya (and several of the other countries included) the urban-rural differences disappeared when socioeconomic status was taken into account [28]. Research conducted on urban-rural differences in childcare practices, has found significant differences in treatment seeking for child fever [32] and child illness [33] favoring urban compared to rural households. Matanda and colleagues found different time trend patterns of breast- and complementary feeding depending on geographic region and urban/rural residence in Kenya [34]. Other studies have also found indications that urban poor children are actually worse off than their rural counterparts [29,30]. The rapid population growth and urbanization might be changing urban-rural or other geographic patterns of childcare as well as the role played by socioeconomic factors in these patterns. Hence, the role of context in a comprehensive analysis including socioeconomic and other background factors is relevant in examining development and status of childcare practices.

Policy makers need such analyses to inform more effective health promotion activities. However in Kenya until now, the studies that have investigated childcare practices have focused on single care practices, and/or used data that are not nationally representative, and/or have not examined patterns of optimal childcare over a period of time [33,35–37]. There is consequently a need for a comprehensive, longitudinal examination of patterns of childcare practices in Kenya, including urban-rural, regional and socioeconomic differences. The aim of this study was to contribute to the knowledge about possible changes in optimal childcare practices in Kenya, with a specific focus on potential differences in childcare between geographic regions and between urban and rural residence. Specifically, the study examined to what extent there has been changes in optimal childcare practices in Kenya using nationally representative surveys conducted in 2003, 2008–9 and 2014.

## Materials and Methods

### Data sources and study samples

The analyses in this study are based on data from Demographic and Health Surveys (DHS) conducted in Kenya in 2003, 2008–9 and 2014. The surveys were implemented by the Kenya National Bureau of Statistics in collaboration with other governmental and non-governmental entities, and with technical assistance from the international MEASURE DHS [2,19,20]. All person-identifying information was removed from these publically-available datasets prior to their release for analysis.

The 2003, 2008–9 and 2014 Kenya Demographic and Health Surveys (KDHS) are nationally representative household-based surveys that utilize a two-stage sampling design. First, data collection points or clusters are selected from the national master frame followed by a systematic sampling of households from the clusters. In the 2003 survey, 400 clusters comprising of 133 urban and 267 rural areas were selected from the master frame while the 2008–9 survey had 400 clusters selected representing 129 urban and 271 rural areas. The 2014 survey was a little different as it was designed to produce representative estimates for most of the survey indicators at the national level, for urban and rural areas separately, at the regional level (since the promulgation of the 2010 constitution, provinces were renamed as regions and 47 counties were created which serve as devolved units of administration), and for selected indicators at the county level. Consequently, more households were sampled in 2014 as compared to previous surveys. Therefore, in the 2014 survey, 1612 clusters were selected representing 617 in urban areas and 995 clusters in rural areas. In the 2003, 2008–9 and 2014 surveys, 9057, 8561 and 36430 household interviews were conducted with 96, 98, and 99 percent response rates respectively. Individual interviews were conducted among women of ages 15–49 years to collect data on both their health and that of their under-five children. A total of 5949, 6079 and 20964 women interviewed in 2003, 2008–9 and 2014 respectively were used in the analysis.

### Measures

Optimal childcare practices are the outcome variables and were classified into two main categories: health facility-based childcare practices and home-based childcare practices.

#### Health facility-based childcare practices

*Complete immunization*: Age specific immunization status whereby children at ages ≤ 1 month received Bacillus Calmette-Guerin (BCG); ≥ 2 and < 4 months received BCG, vaccinated against Diphtheria, Pertussis and Tetanus (DPT1), and Polio (Polio1); ≥ 4 and < 6 months received BCG, DPT1, Polio1, DPT2 and Polio2; ≥ 6 and < 12 months received BCG, DPT1, Polio1, DPT2, Polio2, DPT3 and Polio3; ≥ 12 and ≤ 59 months received BCG, DPT1, Polio1, DPT2, Polio2, DPT3, Polio3 and Measles.

*Antenatal visits*: Mothers visited a health facility four or more times during their pregnancy for antenatal care.

*Medical treatment for fever*: Children taken to a health facility for treatment if they had fever/cough in the course of the past two weeks prior to the survey.

*Medical treatment for diarrhea*: Children taken to a health facility for treatment it they had diarrhea in the course of the past two weeks prior to the survey.

#### Home-based childcare practices

*Use of insecticide-treated bed net (ITNs)*: Children slept under ITNs the night prior to the survey.

*Sanitary disposal of stool*: Children’s stool disposed in a toilet or latrine.

*Use of oral rehydration solutions (ORS) during diarrhea*: Children given a fluid made from a special packet called Oralite/Oral dehydration salts if they had diarrhea in the course of the past two weeks prior to the survey.

*Amount offered to drink during diarrhea*: Children given same amount or more than usual to drink if they had diarrhea in the course of the past two weeks prior to the survey.

*Amount offered to eat during diarrhea*: Children given same amount or more than usual to eat if they had diarrhea in the course of the past two weeks prior to the survey.

*Consumption of minimum acceptable diet*: Children of ages 6–23 months who received a minimum acceptable diet as recommend by WHO [38]. This variable was computed by combining breastfed and non-breastfed children of ages 6–23 months who had at least the minimum recommended dietary diversity and meal frequency. Specifications for breastfed children was that they received foods from 4 or more food groups for their minimum dietary diversity requirement, and received meals 2 times for ages 6–8 months and 3 times for ages 9–23 months for their minimum meal frequency, 24 hours prior to the survey. For non-breastfed children, the condition was that they received foods from 4 or more food groups for their minimum dietary diversity requirement, and that they received meals 4 times for their minimum meal frequency 24 hours prior to the survey. We constructed six food groups: i) Grains, roots and tubers; ii) Legumes and nuts; iii) Dairy products (milk, yogurt, cheese); iv) Animal products (meat, eggs, fish, poultry and liver/organ meats); v) vitamin-A rich fruits and vegetables; vi) other fruits and vegetables. It is important to note that WHO recommends eggs be grouped in its own food category [38] but this was not possible in this study since KDHS combined eggs and other animal products into one variable during data collection.

Predictor variables comprised of socio-economic and demographic factors which included: wealth quintiles using the DHS wealth index WI (a proxy for standard of living based on household ownership of assets and housing quality) [39], maternal education (completion of formal levels of education), religion (Christian or not), residence (living in urban or rural administrative area), radio exposure (media exposure through radio at least once in a week), and maternal literacy (ability to read simple text). Maternal decision making latitude variable referred to a woman’s independence in making household and own health decisions (having a final say on her own healthcare, purchase of large household goods, purchase of daily household goods, visiting friends/relatives, and the type of food to be cooked), while maternal wife beating attitude variable was constructed by considering whether a woman believed that wife beating was justified when she goes out without permission, neglects children, when she argues with the husband, refuses sex with the husband, and burns food. We also constructed a season's variable based on the month the KDHS data was collected and the Kenyan weather pattern: January, February and March (Dry season); April, May and June (Long rains season); and July, August, September, October, November and December (Short rains season).

### Data analysis

**Fig 1:**
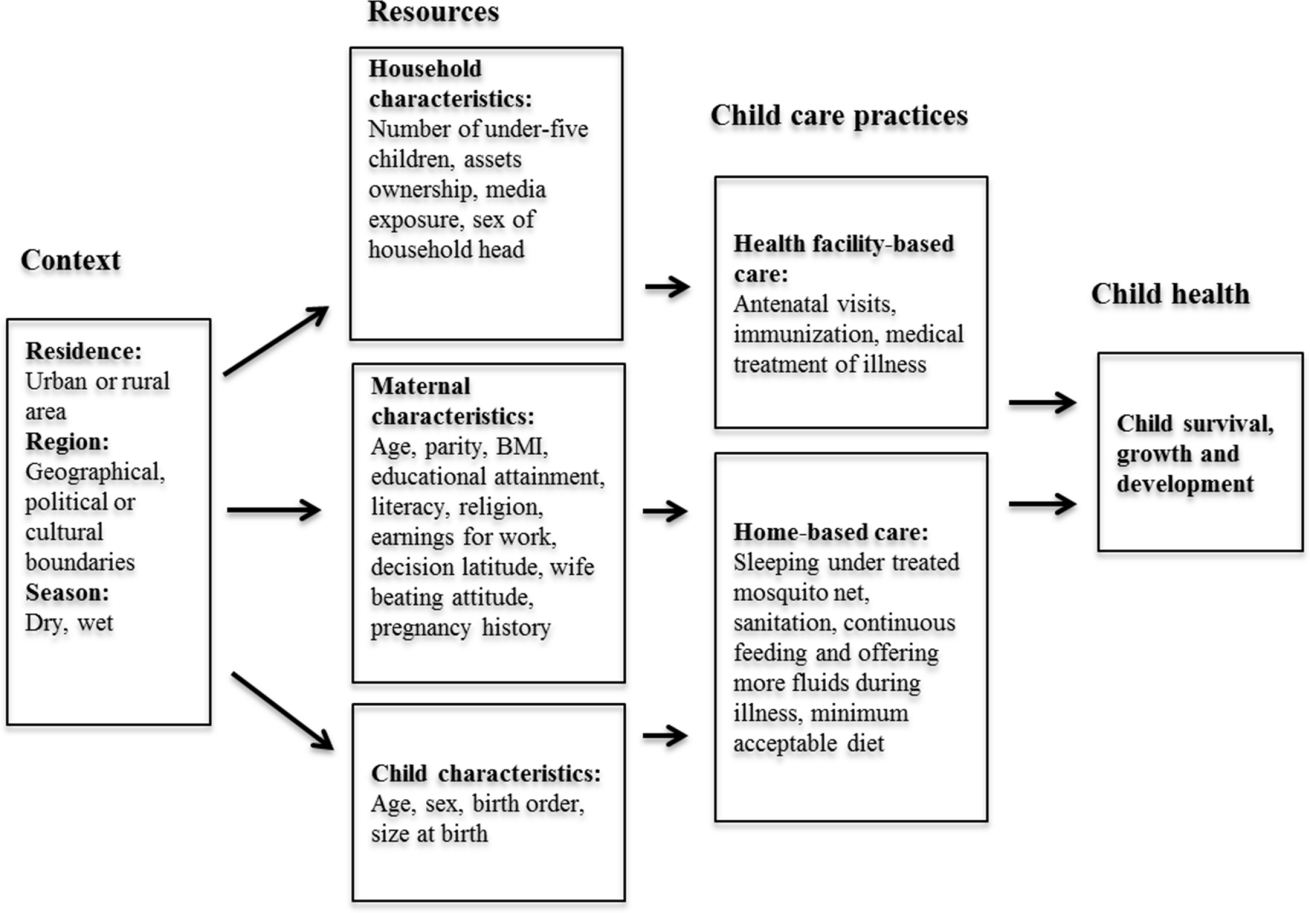
Analytical framework.

Fig 1 is an analytical framework used to guide the analysis. The framework hypothesizes that contextual factors such as place of residence and season affect resources available at the household and individual levels. Resources at the household and individual levels affect the type of care given to children which then affects their health and development. Based on the analytical framework, patterns of optimal childcare practices were ascertained by comparing changes in prevalence of facility- and home-based care between 2003 and 2014. We then used logistic regression to examine correlates of optimal childcare practices in 2003, 2008–9 and 2014. Logit models were organized to first assess the gross effects of place of residence on optimal childcare practices (logit model 1), followed by an assessment of net effects of place of residence on optimal childcare practices adjusting for other socio-economic and demographic variables (logit model 2). To take into account the oversampling of urban areas and the multi-stage KDHS sampling design, SPSS’s complex samples module was used to incorporate sample weight, cluster and strata in all the analyses.

## Results

Characteristics of respondents in the three surveys are given in Table 1. The table shows that there were minimal differences in samples across the three surveys stratified by the different socio-demographic segments. Table 2 shows patterns of facility- and home-based care practices in 2003, 2008–9 and 2014. Comparing the 2003 and 2014 prevalence, there were changes in the prevalence of all childcare practices. However, not all practices increased in prevalence; quality care during child’s bouts of diarrhea and consumption of a minimum acceptable diet actually decreased. Among the childcare practices that increased in prevalence, the largest improvements were in bed net use (home-based care) and medical treatment for diarrhea (facility-based care). Of these, bed net use improved remarkably, from 6% in the 2003 data to 57% in the 2014 data.

**Table 1:** Comparison of the socio-demographic characteristics of KDHS 2003, 2008–9 and the 2014 samples.

	KDHS 2003		KDHS 2008–9		KDHS 2014	
	n	%	n	%	n	%
**Total**	5949	100.0	6079	100.0	20964	100.0
**Child's sex**						
Male	3015	51.0	3134	51.7	10633	50.8
Female	2934	49.0	2945	48.3	10331	49.2
**Region**						
Nairobi	548	6.5	414	5.7	532	10.5
North-Eastern	452	3.0	583	3.0	1594	3.3
Rift-Valley	1200	26.9	1060	28.1	6850	29.0
Nyanza	792	16.4	1109	19.6	2926	14.3
Eastern	700	15.5	744	15.2	3015	11.9
Coast	699	8.4	883	8.5	2650	10.3
Central	730	10.7	496	8.0	1420	9.2
Western	828	12.7	790	12.0	1977	11.5
**Residence**						
Urban	1534	18.7	1467	18.4	6828	35.9
Rural	4415	81.3	4612	81.6	14136	64.1
**Season**						
Dry	936	15.4	3328	55.2	n/a	n/a
Short rains	1554	24.8	2751	44.8	14079	69.1
Long rains	3459	59.8	n/a	n/a	6885	30.9
**Wealth quintiles**						
Richest	1319	18.5	1253	18.7	2810	20.1
Richer	937	16.9	985	17.7	3131	17.7
Middle	1077	19.0	985	18.5	3497	18.0
Poorer	1117	20.8	1079	20.3	4348	20.4
Poorest	1499	24.7	1777	24.7	7178	23.8
**Maternal education**						
Secondary +	1283	20.7	1349	23.5	5324	32.1
Primary	3456	63.9	3430	63.5	1105	56.1
No education	1210	15.4	1300	13.0	4585	11.8
**Pregnancy history**						
Wanted	3153	49.6	3473	50.9	6187	58.1
Mistimed/unwanted	2792	50.4	2602	49.1	3892	41.9
**Size at birth**						
Large	1443	25.4	1873	16.1	2372	25.9
Average	3482	58.2	3080	51.8	5896	58.9
Small	983	16.4	1044	16.1	1660	15.2
**Religion**						
Christian	4807	87.9	4608	87.1	16803	88.7
Non-Christian	1135	12.1	1462	12.9	4123	11.3
**Maternal BMI**						
Normal	3909	69.9	3980	67.8	6192	61.6
Overweight/obese	1043	18.1	1255	20.5	2617	30.0
Underweight	704	12.1	775	11.8	1184	8.5
**Literacy**						
Reads easily	3742	66.1	3575	66.5	12948	71.9
Reads with difficulty	446	8.4	808	14.2	2077	9.8
Cannot read	1746	25.4	1635	19.3	5867	18.3
**Listens to radio**						
Yes	4653	81.4	4664	83.1	7273	43.2
No	1291	18.6	1411	16.9	13677	56.8
**Sex of household head**						
Female	1497	26.1	1775	28.8	6260	27.5
Male	4452	73.9	4304	71.2	14704	72.5
**Earnings for work**						
Paid	2917	78.7	2658	73.2	4995	80.9
Not paid	764	21.3	829	26.8	1403	19.1
**Decision making latitude**						
Independent	512	8.6	243	4.2	581	5.8
Not independent	5426	91.4	4935	95.8	7942	94.2
**Wife beating attitude**						
Not justified	1473	24.5	2578	41.4	4767	53.2
Justified	4431	75.5	3445	58.6	5309	46.8
	**Mean**	**SD**	**Mean**	**SD**	**Mean**	**SD**
**Maternal age (years)**	28.3	6.7	28.3	6.6	28.6	6.6
**Parity**	3.9	2.5	3.8	2.3	3.5	2.3
**Child's age (months)**	27.8	17.4	28.7	17.2	29.1	17.2
**Number of children (≤ 5 years)**	1.8	0.9	1.9	1.0	1.7	0.9

Secondary+, secondary and/or higher education; SD, standard deviation; BMI, body mass index

**Table 2:** National patterns: Health facility- and home-based optimal childcare practices.

	KDHS 2003		KDHS 2008–9		KDHS 2014		Change (2003 vs 2014)
	%	n	%	n	%	n	%
**Health facility-based care**							
Complete immunization	54.6	2745	66.4	3397	72.8	13477	18.2
Antenatal visits (≥ 4)	53.8	2081	48.2	1899	57.8	8093	4.0
Medical treatment for fever	43.3	1255	45.3	915	53.6	4563	10.3
Medical treatment for diarrhea	29.7	258	48.6	475	57.8	1683	28.1
**Home-based care**							
Child slept under treated mosquito net	6.3	359	48.0	2864	56.7	11361	50.4
Child's stool disposal (toilet/latrine)	51.4	2807	69.8	3773	75.1	6618	23.7
Used ORS during diarrhea	29.4	252	72.1	652	54.2	1529	24.8
Same/more fluid offered during diarrhea	68.8	536	57.3	495	58.3	1511	-10.5
Same/more food offered during diarrhea	41.7	318	34.6	300	36.0	918	-5.7
Consumption of a minimum acceptable diet	24.9	393	19.3	267	14.7	311	-10.2

Tables 3–6 show results of logistic regression analyses. Here, it is important to note that due to limited space and to avoid presentation of overwhelming data, we have only presented logit models of optimal childcare practices that showed some consistency in their predictions (statistically significant predictor variables appearing across the three surveys). Detailed logistic regression results for all the other optimal childcare practices are available in S1–S5 Tables.

**Table 3:** Correlates of complxete immunization.

	KDHS 2003		KDHS 2008–9		KDHS 2014	
	OR	95% CI	OR	95% CI	OR	95% CI
**Model 1: Gross effects of place of residence**						
Residence (Ref: Urban)						
- Rural	0.99	0.77–1.29	0.92	0.73–1.18	1.07	0.95–1.20
Region (Ref: Nairobi)						
- North-Eastern	13.75***	7.91–23.91	2.56***	1.59–4.12	4.43***	3.19–6.15
- Rift-Valley	1.47	0.98–2.21	0.64	0.41–1.01	1.30	0.97–1.74
- Nyanza	2.43***	1.54–3.84	1.45	0.93–2.26	1.68***	1.25–2.27
- Eastern	0.99	0.64–1.54	0.47**	0.29–0.76	0.65*	0.46–0.91
- Coast	1.03	0.66–1.62	0.80	0.52–1.21	0.96	0.70–1.33
- Central	0.67	0.44–1.02	0.33***	0.19–0.58	0.94	0.68–1.31
- Western	1.93**	1.23–3.02	0.99	0.59–1.65	1.13	0.81–1.58
**Model 2: Net effects of place of residence controlling for other socio-demographic variables**						
Residence (Ref: Urban)						
- Rural	0.77	0.55–1.09	0.74	0.45–1.21	0.75*	0.58–0.96
Region (Ref: Nairobi)						
- North-Eastern	5.89***	2.10–16.48	1.37	0.37–5.10	2.26	0.92–5.55
- Rift-Valley	1.29	0.78–2.14	0.59	0.30–1.19	1.72	0.89–3.31
- Nyanza	2.83***	1.65–4.85	1.34	0.64–2.79	2.23*	1.15–4.34
- Eastern	1.23	0.72–2.12	0.46*	0.21–0.98	1.07	0.53–2.18
- Coast	1.06	0.63–1.80	0.72	0.32–1.63	1.09	0.53–2.23
- Central	1.04	0.60–1.81	0.44*	0.22–0.88	1.53	0.76–3.06
- Western	2.11**	1.22–3.66	1.19	0.52–2.73	1.37	0.67–2.80
**Season** (Ref: Short rains)						
- Dry	1.22	0.82–1.81	0.86	0.63–1.16	n/a	n/a
- Long rains	1.30	1.00–1.68	n/a	n/a	1.33**	1.08–1.65
**Household characteristics**						
Number of children ages ≤ 5 years	0.91	0.81–1.03	1.06	0.91–1.24	1.19**	1.05–1.35
Wealth quintiles (Ref: Richest)						
- Richer	0.67*	0.47–0.94	0.54*	0.30–0.98	1.02	0.66–1.58
- Middle	0.70	0.49–1.02	0.69	0.40–1.21	1.09	0.71–1.67
- Poorer	0.65*	0.45–0.94	0.73	0.42–1.26	1.22	0.78–1.90
- Poorest	0.76	0.51–1.12	0.84	0.46–1.53	1.50	0.93–2.42
Listens to radio (Ref: Yes)						
- No	1.56***	1.21–2.01	1.60**	1.13–2.27	0.99	0.80–1.23
Sex of household head (Ref: Female)						
- Male	1.13	0.89–1.45	0.94	0.69–1.29	1.13	0.90–1.42
**Maternal/Child's characteristics**						
Mother's age	0.96***	0.93–0.98	0.99	0.97–1.02	0.99	0.96–1.01
Parity	1.14***	1.06–1.22	1.11*	1.01–1.21	1.09**	1.03–1.16
BMI (Ref: Normal)						
- Overweight/obese	0.90	0.71–1.14	0.94	0.67–1.33	0.93	0.75–1.16
- Underweight	1.11	0.84–1.47	1.05	0.71–1.56	0.87	0.60–1.26
Education (Ref: Secondary +)						
- Primary	1.00	0.78–1.27	1.37	0.91–2.05	0.93	0.72–1.21
- No education	1.31	0.83–2.08	1.54	0.73–3.23	1.45	0.89–2.35
Earnings for work (Ref: Paid)						
- Not paid	0.87	0.68–1.11	1.22	0.92–1.61	0.90	0.70–1.15
Literacy (Ref: Reads easily)						
- Reads with difficulty	1.34	0.86–2.09	1.17	0.81–1.70	1.35*	1.02–1.80
- Cannot read	1.40	1.00–1.95	1.45	0.94–2.24	1.29	0.94–1.77
Religion (Ref: Christian)						
- Non-Christian	0.99	0.65–1.51	0.65*	0.43–0.98	1.28	0.86–1.92
Decision making (Ref: Independent)						
- Not independent	0.69	0.48–1.00	1.08	0.64–1.80	0.91	0.66–1.24
Wife beating (Ref: Not justified)						
- Justified	1.33*	1.04–1.71	0.93	0.71–1.22	1.10	0.90–1.35
Pregnancy history (Ref: wanted)						
- Mistimed/unwanted	1.11	0.90–1.36	1.04	0.79–1.37	1.04	0.86–1.25
Child's age	1.00	0.99–1.00	1.00	0.99–1.01	1.01***	1.01–1.02
Child's sex (Ref: Male)						
- Female	1.00	0.84–1.19	1.01	0.81–1.25	1.04	0.87–1.23
Child's size at birth (Ref: Large)						
- Average	0.95	0.78–1.17	0.85	0.65–1.12	0.90	0.74–1.09
- Small	1.11	0.83–1.49	1.32	0.90–1.96	1.07	0.81–1.42

* p < .05

** p < .01

*** p < .001

Secondary+, secondary and higher education.

**Table 4:** Correlates of antenatal visits (≥4).

	KDHS 2003		KDHS 2008–9		KDHS 2014	
	OR	95% CI	OR	95% CI	OR	95% CI
**Model 1: Gross effects of place of residence**						
Residence (Ref: Urban)						
- Rural	1.95***	1.55–2.45	1.81***	1.26–2.60	1.73***	1.57–1.92
Region (Ref: Nairobi)						
- North-Eastern	11.41***	7.42–17.54	2.61**	1.40–4.86	3.23***	2.33–4.49
- Rift-Valley	1.39	0.96–2.02	1.57	0.87–2.81	1.74***	1.37–2.20
- Nyanza	1.77**	1.21–2.60	1.72*	1.05–2.84	1.30*	1.01–1.68
- Eastern	1.93***	1.31–2.83	1.32	0.78–2.24	1.42**	1.10–1.83
- Coast	1.61*	1.12–2.32	1.69*	1.07–2.66	1.23	0.95–1.59
- Central	0.89	0.61–1.29	0.94	0.55–1.59	1.17	0.90–1.51
- Western	1.21	0.81–1.81	1.79*	1.04–3.07	1.65***	1.27–2.14
**Model 2: Net effects of place of residence controlling for other socio-demographic variables**						
Residence (Ref: Urban)						
- Rural	1.14	0.81–1.59	1.00	0.61–1.63	1.13	0.89–1.42
Region (Ref: Nairobi)						
- North-Eastern	4.97***	2.08–11.92	3.03*	1.13–8.11	0.38	0.11–1.30
- Rift-Valley	1.04	0.63–1.73	1.26	0.59–2.68	1.32	0.76–2.30
- Nyanza	1.57	0.93–2.65	1.78	0.87–3.65	0.92	0.51–1.65
- Eastern	2.08**	1.19–3.62	1.35	0.63–2.89	1.01	0.57–1.80
- Coast	0.98	0.58–1.66	1.22	0.59–2.50	0.57	0.29–1.12
- Central	0.95	0.56–1.63	1.46	0.70–3.02	1.52	0.86–2.70
- Western	0.91	0.53–1.57	1.22	0.56–2.66	1.06	0.60–1.90
**Season** (Ref: Short rains)						
- Dry	0.84	0.59–1.20	0.85	0.65–1.12	n/a	n/a
- Long rains	0.78	0.61–1.01	n/a	n/a	0.97	0.79–1.18
**Household characteristics**						
Number of children ages ≤ 5 years	1.10	0.98–1.23	1.23*	1.04–1.45	1.10	0.96–1.25
Wealth quintiles (Ref: Richest)						
- Richer	1.27	0.82–1.97	1.14	0.72–1.81	1.51*	1.01–2.25
- Middle	1.25	0.78–1.99	1.65	0.97–2.82	1.71*	1.13–2.60
- Poorer	1.50	0.98–2.31	2.25***	1.37–3.68	1.90**	1.23–2.95
- Poorest	1.37	0.85–2.21	1.35	0.83–2.21	2.00**	1.27–3.15
Listens to radio (Ref: Yes)						
- No	1.00	0.75–1.33	1.32	0.85–2.04	1.09	0.87–1.36
Sex of household head (Ref: Female)						
- Male	0.91	0.71–1.17	1.06	0.79–1.43	1.04	0.85–1.27
**Maternal/Child's characteristics**						
Mother's age	0.96**	0.93–0.98	0.97*	0.94–0.99	0.97**	0.94–0.99
Parity	1.15***	1.06–1.25	1.09	0.98–1.21	1.14***	1.06–1.23
BMI (Ref: Normal)						
- Overweight/obese	0.85	0.64–1.13	1.07	0.78–1.49	0.96	0.77–1.19
- Underweight	1.34	0.98–1.82	1.07	0.73–1.58	0.98	0.69–1.39
Education (Ref: Secondary +)						
- Primary	1.29*	1.01–1.66	1.45*	1.09–1.94	1.36*	1.07–1.74
- No education	1.75*	1.12–2.74	1.61	0.79–3.27	2.37***	1.40–4.01
Earnings for work (Ref: Paid)						
- Not paid	0.91	0.68–1.22	1.04	0.78–1.40	1.07	0.88–1.30
Literacy (Ref: Reads easily)						
- Reads with difficulty	2.10***	1.36–3.26	1.54	1.00–2.37	0.87	0.67–1.15
- Cannot read	1.10	0.78–1.55	1.67	0.99–2.80	0.85	0.61–1.17
Religion (Ref: Christian)						
- Non-Christian	1.46	0.99–2.16	1.27	0.83–1.95	1.68*	1.07–2.62
Decision making (Ref: Independent)						
- Not independent	1.02	0.71–1.45	1.06	0.66–1.69	1.14	0.85–1.52
Wife beating (Ref: Not justified)						
- Justified	1.10	0.86–1.40	1.05	0.79–1.39	1.00	0.83–1.20
Pregnancy history (Ref: wanted)						
- Mistimed/unwanted	1.53***	1.28–1.84	1.51***	1.18–1.93	1.32**	1.11–1.58

* p < .05

** p < .01

*** p < .001

Secondary+, secondary and higher education.

**Table 5:** Correlates of insecticide treated bed net use.

	KDHS 2003		KDHS 2008–9		KDHS 2014	
	OR	95% CI	OR	95% CI	OR	95% CI
**Model 1: Gross effects of place of residence**						
Residence (Ref: Urban)						
- Rural	3.30***	2.12–5.16	2.67***	1.99–3.59	1.37***	1.19–1.56
Region (Ref: Nairobi)						
- North-Eastern	3.22***	1.15–8.99	0.27***	0.15–0.50	1.15	0.78–1.69
- Rift-Valley	1.34	0.69–2.60	1.08	0.63–1.85	0.94	0.70–1.27
- Nyanza	0.41**	0.21–0.77	0.29***	0.17–0.49	0.26***	0.19–0.36
- Eastern	0.75	0.35–1.60	0.42**	0.24–0.74	0.60***	0.44–0.82
- Coast	0.65	0.34–1.24	0.45**	0.27–0.75	0.36***	0.26–0.50
- Central	0.75	0.37–1.50	0.92	0.50–1.71	1.19	0.83–1.70
- Western	0.49*	0.26–0.93	0.31***	0.18–0.53	0.23***	0.16–0.32
**Model 2: Net effects of place of residence controlling for other socio-demographic variables**						
Residence (Ref: Urban)						
- Rural	0.71	0.34–1.50	1.79*	1.09–2.94	0.84	0.64–1.09
Region (Ref: Nairobi)						
- North-Eastern	2.04***	1.01–4.11	0.25**	0.09–0.65	0.08***	0.02–0.25
- Rift-Valley	0.61	0.26–1.47	0.56	0.28–1.16	0.54*	0.32–0.90
- Nyanza	0.15***	0.07–0.35	0.16***	0.08–0.33	0.14***	0.08–0.24
- Eastern	0.26**	0.11–0.61	0.34**	0.16–0.72	0.36***	0.21–0.61
- Coast	0.22***	0.10–0.46	0.25***	0.12–0.53	0.17***	0.09–0.32
- Central	0.60	0.22–1.63	0.91	0.44–1.89	1.43	0.81–2.55
- Western	1.19***	0.09–0.42	0.19***	0.09–0.40	0.11***	0.06–0.20
**Season** (Ref: Short rains)						
- Dry	0.78	0.39–1.54	0.84	0.63–1.12	n/a	n/a
- Long rains	0.73	0.43–1.24	n/a	n/a	0.84	0.66–1.06
**Household characteristics**						
Number of children ages ≤ 5 years	1.04	0.82–1.31	1.17	0.99–1.39	0.87	0.75–1.01
Wealth quintiles (Ref: Richest)						
- Richer	2.42*	1.20–4.88	1.37	0.81–2.31	1.37	0.92–2.05
- Middle	3.03**	1.38–6.68	1.29	0.77–2.15	1.57*	1.04–2.38
- Poorer	8.86***	3.98–19.75	1.97**	1.18–3.30	1.76*	1.14–2.72
- Poorest	14.82***	4.74–46.38	2.11*	1.11–4.00	2.25***	1.40–3.61
Listens to radio (Ref: Yes)						
- No	1.49	0.82–2.73	1.33	0.90–1.97	1.22	0.96–1.54
Sex of household head (Ref: Female)						
- Male	1.07	0.68–1.69	0.87	0.61–1.23	0.80	0.64–1.01
**Maternal/Child's characteristics**						
Mother's age	0.98	0.93–1.03	0.98	0.95–1.02	0.97**	0.94–0.99
Parity	1.08	0.93–1.25	1.09	0.99–1.19	1.14***	1.06–1.23
BMI (Ref: Normal)						
- Overweight/obese	0.76	0.51–1.14	1.00	0.75–1.34	0.93	0.74–1.17
- Underweight	0.73	0.40–1.34	1.12	0.77–1.65	0.87	0.60–1.27
Education (Ref: Secondary +)						
- Primary	2.12***	1.40–3.23	1.22	0.88–1.70	1.24	0.97–1.60
- No education	1.95	0.57–6.68	1.27	0.52–3.11	3.15***	1.78–5.58
Earnings for work (Ref: Paid)						
- Not paid	0.98	0.63–1.53	0.96	0.67–1.36	0.92	0.71–1.20
Literacy (Ref: Reads easily)						
- Reads with difficulty	1.86	0.63–5.45	1.15	0.77–1.71	1.45	1.00–2.09
- Cannot read	1.48	0.66–3.34	0.91	0.54–1.51	0.87	0.59–1.29
Religion (Ref: Christian)						
- Non-Christian	1.10	0.54–2.22	0.80	0.49–1.31	1.28	0.79–2.07
Decision making (Ref: Independent)						
- Not independent	0.62	0.31–1.27	0.62	0.38–1.01	0.99	0.69–1.42
Wife beating (Ref: Not justified)						
- Justified	1.02	0.70–1.49	1.10	0.83–1.44	0.87	0.70–1.09
Pregnancy history (Ref: wanted)						
- Mistimed/Unwanted	1.54*	1.06–2.23	0.93	0.69–1.25	1.12	0.90–1.39
Child's age	1.00	0.99–1.01	1.02***	1.01–1.02	1.01***	1.01–1.01
Child's sex (Ref: Male)						
- Female	0.93	0.64–1.36	1.01	0.82–1.25	1.19*	1.02–1.38
Child's size at birth (Ref: Large)						
- Average	1.07	0.72–1.59	0.81	0.64–1.03	0.87	0.70–1.07
- Small	1.08	0.61–1.90	1.04	0.75–1.45	1.07	0.81–1.41

* p < .05

** p < .01

*** p < .001

Secondary+, secondary and higher education.

**Table 6:** Correlates of disposal of child's stool in a toilet/latrine.

	KDHS 2003		KDHS 2008–9		KDHS 2014	
	OR	95% CI	OR	95% CI	OR	95% CI
**Model 1: Gross effects of place of residence**						
Residence (Ref: Urban)						
- Rural	2.35***	1.71–3.23	6.15***	3.23–11.72	4.13***	3.25–5.23
Region (Ref: Nairobi)						
- North-Eastern	5.79***	2.19–15.36	18.14***	6.08–54.08	1.58	0.84–2.97
- Rift-Valley	1.07	0.70–1.65	4.25**	1.44–12.52	0.39***	0.23–0.65
- Nyanza	0.67	0.41–1.10	3.50*	1.16–10.55	0.27***	0.16–0.48
- Eastern	0.44***	0.28–0.69	2.19	0.70–6.86	0.17***	0.09–0.32
- Coast	1.41	0.85–2.35	8.64***	3.20–23.30	0.63	0.37–1.07
- Central	0.66	0.42–1.02	0.51	0.14–1.81	0.06***	0.03–0.12
- Western	0.35***	0.21–0.57	0.71	0.21–2.37	0.11***	0.06–0.20
**Model 2: Net effects of place of residence controlling for other socio-demographic variables**						
Residence (Ref: Urban)						
- Rural	0.89	0.60–1.31	1.02	0.50–2.07	1.41	0.97–2.05
Region (Ref: Nairobi)						
- North-Eastern	1.34	0.25–7.12	4.22	0.41–43.57	0.08***	0.02–0.36
- Rift-Valley	0.76	0.45–1.30	8.32*	1.62–42.72	0.20***	0.09–0.41
- Nyanza	0.46*	0.25–0.86	7.14*	1.35–37.86	0.22***	0.10–0.48
- Eastern	0.52*	0.29–0.95	6.87*	1.20–39.32	0.13***	0.06–0.30
- Coast	0.86	0.46–1.62	8.95*	1.61–49.88	0.18***	0.08–0.43
- Central	0.97	0.57–1.64	1.92	0.33–11.15	0.12***	0.05–0.31
- Western	0.28***	0.14–0.53	1.91	0.32–11.41	0.10***	0.04–0.23
**Season** (Ref: Short rains)						
- Dry	1.00	0.61–1.66	0.93	0.57–1.50	n/a	n/a
- Long rains	1.13	0.84–1.52	n/a	n/a	0.83	0.60–1.15
**Household characteristics**						
Number of children ages ≤ 5 years	1.25*	1.05–1.50	1.00	0.76–1.30	1.26*	1.02–1.56
Wealth quintiles (Ref: Richest)						
- Richer	1.53	0.93–2.51	1.85	0.71–4.84	0.96	0.48–1.91
- Middle	1.48	0.88–2.47	3.69**	1.42–9.61	0.82	0.39–1.73
- Poorer	1.72*	1.05–2.85	4.99***	1.91–13.02	1.52	0.75–3.10
- Poorest	3.79***	2.18–6.58	9.21***	3.48–24.38	5.27***	2.53–11.00
Listens to radio (Ref: Yes)						
- No	0.95	0.68–1.31	1.47	0.91–2.38	1.20	0.85–1.70
Sex of household head (Ref: Female)						
- Male	1.03	0.79–1.36	1.14	0.74–1.77	1.12	0.81–1.55
**Maternal/Child's characteristics**						
Mother's age	0.94***	0.91–0.97	0.94**	0.90–0.98	0.95**	0.92–0.83
Parity	1.19***	1.08–1.31	1.23***	1.09–1.40	1.13*	1.02–1.26
BMI (Ref: Normal)						
- Overweight/obese	0.81	0.60–1.09	0.68	0.39–1.19	1.06	0.76–1.46
- Underweight	1.08	0.71–1.66	1.67*	1.01–2.78	1.44	0.97–2.16
Education (Ref: Secondary +)						
- Primary	1.12	0.79–1.60	1.64	0.98–2.74	0.84	0.58–1.22
- No education	2.54**	1.33–4.87	3.93**	1.55–10.01	2.69**	1.39–5.21
Earnings for work (Ref: Paid)						
- Not paid	1.08	0.83–1.41	1.02	0.72–1.44	1.80***	1.30–2.48
Literacy (Ref: Reads easily)						
- Reads with difficulty	0.72	0.45–1.14	0.92	0.55–1.55	1.93***	1.33–2.81
- Cannot read	0.93	0.62–1.38	1.03	0.57–1.84	0.90	0.56–1.45
Religion (Ref: Christian)						
- Non-Christian	1.24	0.81–1.91	1.36	0.71–2.62	1.28	0.74–2.23
Decision making (Ref: Independent)						
- Not independent	1.09	0.71–1.66	0.72	0.37–1.40	1.07	0.66–1.74
Wife beating (Ref: Not justified)						
- Justified	1.16	0.88–1.53	0.94	0.67–1.34	1.27	0.95–1.70
Pregnancy history (Ref: wanted)						
- Mistimed/Unwanted	1.06	0.84–1.33	0.83	0.54–1.30	1.02	0.77–1.35
Child's age	0.97***	0.96–0.97	0.99***	0.98–1.00	0.98***	0.97–0.99
Child's sex (Ref: Male)						
- Female	0.92	0.78–1.09	0.85	0.66–1.08	1.18	0.95–1.41
Child's size at birth (Ref: Large)						
- Average	0.84	0.68–1.04	1.02	0.74–1.39	0.96	0.71–1.28
- Small	0.97	0.73–1.28	1.24	0.81–1.90	0.79	0.55–1.15

* p < .05

** p < .01

*** p < .001

Secondary+, secondary and higher education.

Table 3 shows logistic regression results for correlates of complete child immunization. Examining the two logit models, region of residence persisted as a significant predictor of a child’s complete immunization status across the three surveys. Children residing in Nyanza region were two times more likely not to be fully immunized in 2003 (OR = 2.8, p < 0.001) and in 2014 (OR = 2.2, p < 0.05) as compared to children in Nairobi. Another significant predictor of child immunization was maternal parity whereby an increase in parity was associated with an increase in the odds of children not achieving complete immunization status: 2003 (OR = 1.1, p < 0.001), 2008–9 (OR = 1.1, p < 0.05) and 2014 (OR = 1.1, p < 0.01).

Table 4 shows the raw and net effects of place of residence as a predictor of mothers attending at least four antenatal care (ANC) visits during their pregnancy. The unadjusted model shows that place of residence is a strong predictor of attending at least four ANC visits but its ability to predict ANC attendance weakens when other socio-demographic variables are adjusted. The adjusted model shows that household wealth, mother’s age, parity, maternal education and pregnancy history are significant predictors of attending at least four ANC visits. Women in poorer wealth quintile were two times more likely not to attend at least four ANC visits in 2008–9 (OR = 2.3, p < 0.001) and in 2014 (OR = 1.9, p < 0.01) as compared to women in the richest wealth quintile. Older women were more likely to attend at least four ANC visits with increase in age being associated with a decrease in the likelihood of not attending at least four ANC visits: 2003 (OR = 0.96, p < 0.01), 2008–9 (OR = 0.97, p < 0.05) and 2014 (OR = 0.97, p < 0.01). An increase in parity on the other hand was associated with an increase in the likelihood of not attending at least four ANC visits in 2003 (OR = 1.2, p < 0.001) and in 2014 (OR = 1.1, p < 0.001). In regard to maternal education, mothers with only some or complete primary education had higher odds of not attending four ANC visits in 2003 (OR = 1.3, p < 0.05), 2008–9 (OR = 1.5, p < 0.05) and 2014 (OR = 1.4, p < 0.05) as compared to mothers with secondary or higher education. Another predictor of ANC attendance was maternal pregnancy history whereby compared to mothers whose pregnancy was wanted, mothers whose pregnancy was mistimed or unwanted were at a higher risk of not attending the recommended four or more ANC visits: 2003 (OR = 1.5, p < 0.001), 2008–9 (OR = 1.5, p < 0.001) and 2014 (OR = 1.3, p < 0.01).

The gross and net effects of place of residence as a predictor of whether a child slept under an insecticide treated bed net (ITN) is shown in Table 5. Examining logit models 1 and 2 show that region of residence is a consistent significant predictor of ITN use in both the unadjusted and adjusted logit models. On the other hand, the effect of urban/rural residence in predicting ITN use was only strong in the unadjusted logit model but weakened when other sociodemographic variables were introduced in the regression equation. Controlling for other socio-demographic variables, children residing in almost all regions in Kenya were more likely to sleep under an insecticide treated bed net in 2008–9 and 2014 as compared to those residing in Nairobi.

Other significant determinants of ITN use were household wealth and child’s age. Children from poor households were significantly at a higher risk of not sleeping under an insecticide treated bed net. For example, comparing the richest against the poorest households shows that children in the poorest households had consistently higher odds of not sleeping under an insecticide treated bed net in 2003 (OR = 14.8, p < 0.001), 2008–9 (OR = 2.1, p < 0.05) and 2014 (OR = 2.3, p < 0.001). In relation to child’s age, the 2008–9 and 2014 data showed that an increase in the child’s age was associated with an increase in the odds of not sleeping under an insecticide treated bed net: 2008–9 (OR = 1.02, p < 0.001) and 2014 (OR = 1.01, p < 0.001).

Table 6 shows logistic regression findings of the correlates of sanitary disposal of child's stool. Logit models 1 and 2 show that region of residence was a consistent predictor of sanitary disposal of a child’s stool. In 2014, households in all the other regions in Kenya were more likely to dispose their children’s stool in a toilet/latrine than households in Nairobi. Of significance is the change in odds between 2008–9 and 2014 in Rift Valley, Nyanza, Eastern and Coast regions. As compared to Nairobi region, households in the aforementioned regions were more likely not to dispose their children’s stool in a toilet/latrine in 2008–9 but this changed in 2014 whereby they were now more likely to dispose their children’s stool in a toilet/latrine with households in Nairobi as a reference.

Household wealth, maternal age, parity, educational attainment, and child’s age were other significant predictors of sanitary disposal of child’s stool. Starting with household wealth, households in the poorest wealth quintile were more likely to engage in unsanitary disposal of their children’s stool as compared to households in the richest wealth quintile: 2003 (OR = 3.8, p < 0.001), 2008–9 (OR = 9.2, p < 0.001) and 2014 (OR = 5.3, p < 0.001). An increase in maternal age was associated with a decrease in unsanitary disposal of a child’s stool in 2003 (OR = 0.94, p < 0.001), 2008–9 (OR = 0.94, p < 0.01) and 2014 (OR = 0.95, p < 0.01). On the other hand, an increase in maternal parity was associated with an increase in the odds of not disposing a child’s stool in a toilet/latrine in 2003 (OR = 1.2, p < 0.001), 2008–9 (OR = 1.2, p < 0.001) and 2014 (OR = 1.1, p < 0.05). With regard to maternal education, mothers with no education were more likely to engage in unsanitary disposal of their children’s stool than those who had attained secondary or higher education: 2003 (OR = 2.5, p < 0.01), 2008–9 (OR = 3.9, p < 0.01) and 2014 (OR = 2.7, p < 0.01). Lastly, an increase in a child’s age was associated with a decrease in unsanitary disposal of the child’s stool in 2003 (OR = 0.97, p < 0.001), 2008–9 (OR = 0.99, p < 0.001) and 2014 (OR = 0.98, p < 0.001).

## Discussion

The study examined patterns of optimal childcare practices in Kenya using surveys conducted in 2003, 2008–9 and 2014. We hypothesized that given the continuous child health promotion activities conducted by the government and by non-government organizations throughout Kenya, the prevalence of health promoting childcare practices would increase significantly during the 11-year period. Study findings at the national level showed that all facility-based childcare practices increased between 2003 and 2014. In relation to home-based care, increases in the use of ITNs, proper disposal of child’s stool and use of ORS during diarrhea were noted. Nonetheless, negative patterns in childcare practices were also experienced with decreases in the proportion of children who were offered same/more fluid/food when they had diarrhea and children who consumed a minimum acceptable diet. With regard to possible determinants of childcare practices, the study noted that area of residence (region), household wealth, maternal education, parity, mother's age, child’s age and pregnancy history were significant correlates of optimal childcare practices in Kenya. In the text that follows, we first discuss some of the health promotion activities that may have informed the observed changes in the national prevalence of optimal childcare practices, and later discuss the significance of the regression findings on the correlates of childcare practices.

The increases in complete immunization, attendance of at least four ANC visits, medical treatment of diarrhea and fever, use of ITNs, proper disposal of child’s stool, and use of ORS during diarrhea could indicate a success story for the various health promotion activities undertaken by the government in collaboration with non-governmental partners. In early 2003, the Kenyan government embarked on a five-year reform strategy in the health sector. There were specific targets geared towards increasing immunization coverage, reducing child mortality and morbidity, increasing access to safe and improved sanitation standards [19]. One notable initiative was the extensive marketing of subsidized ITNs from 2002 to 2004, and later free issuance of ITNs coupled with educational campaigns targeting pregnant women and children under-five [40].

In 2001, Kenya was among a host of countries that benefited from financial support by the Global Alliance for Vaccines and Immunization (GAVI) that aimed at introducing new vaccines and strengthening immunization services. The support was specifically directed towards expansion of vaccine clinics, training of health workers and community mobilization, all geared towards reducing cost and increasing immunization coverage [21,41]. An evaluation study on the coverage of measles vaccine delivered through the supplemental immunization activity from 2001 to 2005 showed that the program reached a greater percentage of previously unvaccinated children [42]. Improvements in the use of ORS, medical treatment for diarrhea/fever and proper disposal of children’s stool could be a result of the government’s efforts to ensure that caregivers are educated on the prevention and management of diarrhea and other childhood illnesses, as highlighted in policy guideline on the control and management of diarrheal diseases in children under-five [43].

Another notable development that could explain national increases in some of the optimal childcare practices, especially health facility-based childcare, are the changes in user fee policy in Kenya [25,26]. In 2004, the Kenyan government removed all user fees at public primary healthcare facilities (dispensaries and health centers), except for a minimum registration fee of 10 or 20 Kenya shillings. There was also a special provision for children under age five and clients with specific health conditions, including malaria and tuberculosis to be exempted from registration fees. In 2007, the government abolished all fees for deliveries in public health facilities and in 2013 it set aside a budget for compensation to lower-level facilities for providing free maternity services [25,26]. These policy changes could have eased the costs incurred by mothers when accessing health services and hence led to the observed increases in facility-based childcare practices.

Despite the encouraging childcare patterns just discussed, attention needs to be given to the decreasing prevalence in the proportion of children who received same/more fluid/food during diarrhea, and children who consumed the minimum acceptable diet. Notwithstanding increases in ORS use, it is perplexing that patterns of home treatment for diarrhea through offering same/more fluids/food declined over the study period. Studies have documented inadequate home-based management of diarrhea in Kenyan households that are clouded with confusion between ORS and other Oral Rehydration Therapies (ORT) [44,45]. Perhaps because of this confusion, it has been reported that most caregivers preferred offering ORS to other ORT and offered much less fluid and food to their children during diarrhea [44]. The present study presents a nuanced analysis, showing that during a period of increased use of ‘technical’ solutions to diarrhea (medical care and ORS), the use of ‘home’ solutions waned. This could be due to a perverse and unintended result of the health campaigns. Even if not saying so explicitly, an implicit message could have been promulgated that ‘medicine is best’.

Results of the present study on child feeding are alarming as they not only show that very few children in Kenya are consuming the minimum acceptable diet, but also that the proportion has significantly reduced from the 2003 to the 2014 survey. This finding is of great public health concern as it comes in the wake of a recent study that showed that trends in child growth in Kenya have either stagnated or gotten worse from 1993 to 2009 [31]. Given that dietary diversity and food frequency are among the proximate determinants of child growth and development [4,46] poor patterns in child consumption of minimum acceptable diet paint a grim picture for efforts to improve child health in Kenya. Research on the food fed to Kenyan children has shown that the food lacks in quantity and more so in quality [47,48]. Various government food policies seem to be falling short, despite laudable aims to support food self-sufficiency and more equitable distribution of quality food to the population [23].

This study hypothesized that health promotion activities between 2003 and 2014 would contribute to significant increases in the prevalence of health promoting childcare practices in Kenya. Yet, many factors other than health promotion might have been at play [49]. Period effects include the health promotion activities listed in the introduction (and many that are not listed), and the myriad of other happenings that influenced Kenyan life from 2003 to 2014. These include changes in the economic and political situations, changes in medical practice and the availability of health services, population composition changes due to in- and-out migration, and social changes due to evolving communications possibilities, evolving norms and changing social lifestyles. It is futile to attempt to precisely attribute social change (such as in childcare practices) to particular period phenomena (such as campaigns). Intertwined with period effects are cohort effects: the children born into the cohort of the 2003 survey did not have the same life exposure experience, opportunities and hindrances as the children born into the cohort of the 2008–9 and 2014 surveys.

We conducted a regression analysis using socio-economic and demographic variables to unravel some of the factors informing the observed national changes in prevalence of some of the childcare practices. Our study findings showed that region, household wealth, maternal education, parity, mother's age, child’s age and pregnancy history were significant correlates of optimal childcare practices. Beginning with region of residence, this study has demonstrated that there are significant regional differences in how children are cared for in Kenya. It is clear that while national patterns show improvements in children achieving complete immunization status, children in Nyanza were at a higher risk of not reaching such a feat. Regional differences were also noted in ITNs use and proper disposal of children’s stool. In 2014, children in almost all the other regions were more likely to have slept under an ITN and their stool properly disposed than children in Nairobi; contrasting the scenario in the earlier surveys where Nairobi was doing better than the other regions. There is a possibility that health promotion campaigns to encourage use of ITNs, proper disposal of child’s stool, and complete immunization did not yield uniform results across the country and that other contextual issues in Nairobi and Nyanza regions need to be investigated. Regional differences in other childcare practices such as breastfeeding have also been noted in other studies in Kenya [34,50,51] and have highlighted existence of complex array of factors including cultural and socioeconomic that come into play when providing care to children.

Turning to household wealth and maternal education as significant correlates of childcare practices, study findings corroborate what has been documented in other studies whereby children of mothers from lower socioeconomic status and educational background tend to receive suboptimal care [33,52–54]. While improvements have been noted in the national prevalence of women attending at least four ANC visits, children sleeping under ITNs and proper disposal of children’s stool, results from the regression analysis showed that poverty and lower educational attainment are still barriers towards attaining optimal childcare practices in Kenya. This could therefore mean that changes in maternal education and household poverty in the 11-year period, both as markers of period phenomena and health promotion activities, was not significant enough to ensure that children in the lower socioeconomic class receive optimal care.

Other significant covariates of childcare practices were maternal age, parity, pregnancy history and child’s age. An increase in maternal age was associated with an increase in the likelihood of attending the recommended four or more ANC visits and proper disposal of child’s stool. Studies that have investigated maternal age and childcare practices have shown that due to poor health seeking behaviors, younger mothers were at a higher risk of receiving inadequate care during pregnancy and were less likely to provide optimal care to their children [52,55]. It is probable that due to the experience that comes with age, older women would likely observe better sanitation at home than the younger unexperienced women.

In relation to maternal parity, women with many births were associated with poor childcare practices such as failure to attend at least four ANC visits, incomplete immunization status of children and poor disposal of child’s stool. It has been argued that multiparous women are likely to have extensive experience of the changes during the pregnancy and childcare and thus less likely to pay attention to practicing healthy behaviors as compared to primiparous mothers who are anxious and more likely to seek maternal and child health services [55,56].

With regard to pregnancy history, mistimed or unwanted pregnancies were associated with poor antenatal care. In a study that investigated the relationship between pregnancy history and receipt of childcare practices in Bolivia, Egypt, Kenya, Peru, and the Philippines, Marston et al., found a significant systematic association between unwanted pregnancy history and poor antenatal care across the five countries [57]. Wanting or planning pregnancy affects a woman's recognition of pregnancy symptoms which determines her early or late entry into prenatal care. Thus, pregnancies that are mistimed or wanted are more likely to be recognized later with fewer visits to the health facility [56,58].

On the relationship between child’s age and childcare practices, higher child age was associated with poor ITNs use but better sanitary stool disposal. Investigating intra-household ITNs use in Ethiopia, Ghana, Mali, Nigeria, Senegal, and Zambia, Baume and Marin [59] found that younger children, especially those under 2 years, were more likely to sleep under a net because they were considered more vulnerable to malaria than older children. On child stool disposal, it has been documented that the use of latrines or toilets is not considered appropriate for younger children until they are three to four years old [60]. Therefore, it is logical that households with older children are likely to observe sanitary disposal of stool as compared to households with younger children.

This study has strengths and limitations. The major strength relates to the use of nationally representative data that enabled national estimates of prevalence and correlates of optimal childcare practices in 2003, 2008–9 and 2014. By studying a combination of childcare practices, this study embraced the importance of conceptualizing care for children as a continuum that spans from inception throughout a child’s life rather than a single event [8]. The major limitation of this study is the use of retrospective, self-reported behavior that is subject to recall bias [61,62]. Nonetheless, the use of maternal reports as a method of data collection is widely used in survey research despite some limitations [63,64].

## Conclusions

We examined changes in facility- and home-based optimal childcare practices in Kenya using data from 2003, 2008–9 and 2014. Between 2003 and 2014, increases were observed in the proportions of pregnant women who attended four or more ANC visits, complete immunization, medical treatment of diarrhea and fever, use of ORS during diarrhea, use of ITNs, and proper disposal of child’s stool. To the contrary, decreases were observed among children who were offered same/more fluid/food when they had diarrhea and children who consumed the minimum acceptable diet. Area of residence (region), household wealth, maternal education, parity, mother's age, child’s age and pregnancy history were significant determinants of optimal childcare practices across the three surveys.

National, regional and local child health promotion activities, coupled with changes in society and in living conditions between 2003 and 2014, could have influenced uptake of certain recommended childcare practices in Kenya, while other practices worsened. Concerted efforts are needed to address the consistent inequities in the uptake of the recommended childcare practices. Such efforts should be cognizant of the underlying factors that affect childcare in Kenya such as region of residence, household wealth, maternal education, parity, mother's age, child’s age and pregnancy history. Also important is linking child health education promotion activities that are informed by recent advances in health communication scholarship [65,66].

## Supporting Information

S1 Table Logistic regression results of determinants of complete immunization and antenatal visits (≥4). (XLSX)

S2 Table Logistic regression results of determinants of medial treatment for fever and diarrhea. (XLSX)

S3 Table Logistic regression results of determinants of insecticide treated bednets use and sanitary disposal of child’s stools. (XLSX)

S4 Table Logistic regression results of determinants of amount offered to drink and eat during diarrhea. (XLSX)

S5 Table Logistic regression results of determinants of oral rehydration salts use during diarrhea and consumption of a minimum acceptable diet. (XLSX)

## References

[1] UN. The Millennium Development Goals Report 2015. New York: United Nations; 2015.

[2] KNBS, ICF International. Kenya Demographic and Health Survey 2014. Calverton, Maryland: KNBS & ICF International; 2014.

[3] Grantham-McGregor S, Cheung YB, Cueto S, Glewwe P, Richter L, Strupp B. Developmental potential in the first 5 years for children in developing countries. The Lancet. 2007 1 12;369(9555):60–70. 10.1016/S0140-6736(07)60032-4 PMC2270351 17208643

[4] Engle PL, Menon P, Garrett JL, Slack A. Urbanization and Caregiving: A Framework for Analysis and Examples from Southern and Eastern Africa. Environ Urban. 1997 10 1;9(2):253–70.

[5] Engle PL, Menon P, Haddad LJ. Care and Nutrition: Concepts and Measurement. Intl Food Policy Res Inst; 1997. 60 p.

[6] Matanda DJ. Child Physical Growth and Care Practices in Kenya: Evidence from Demographic and Health Surveys. Bergen: The University of Bergen; 2015 Available: https://bora.uib.no/handle/1956/9606. Accessed 19 June 2016.

[7] Smith LC, Haddad LJ. Explaining child malnutrition in developing countries: a cross-country analysis. Intl Food Policy Res Inst; 2000. 256 p.

[8] Kerber KJ, de Graft-Johnson JE, Bhutta ZA, Okong P, Starrs A, Lawn JE. Continuum of care for maternal, newborn, and child health: from slogan to service delivery. The Lancet. 2007;370(9595):1358–1369. 10.1016/S0140-6736(07)61578-5 17933651

[9] Lawn J, Kerber K. Opportunities for Africas newborns: practical data policy and programmatic support for newborn care in Africa. 2006; Available: http://www.popline.org/node/179907. Accessed 6 June 2016.

[10] Hill Z, Kirkwood B, Edmond K. Family and community practices that promote child survival, growth and development. Geneva WHO. 2004; Available: http://www.coregroup.org/storage/documents/CCM/who_keyfamilypracticesevidence.pdf. Accessed 6 June 2016.

[11] Boerma JT, Bryce J, Kinfu Y, Axelson H, Victora CG. Mind the gap: equity and trends in coverage of maternal, newborn, and child health services in 54 Countdown countries. Lancet. 2008;371(9620):1259–1267. 10.1016/S0140-6736(08)60560-7 18406860

[12] AbouZahr C, Wardlaw T. Antenatal care in developing countries: promises, achievements and missed opportunities-an analysis of trends, levels and differentials, 1990–2001. World Health Organization; 2003. Accessed 6 June 2016.

[13] UNICEF. The state of the world’s children 2009: maternal and newborn health. Vol. 9 Unicef; 2008. Accessed 17 June 2016.

[14] Hawley WA, Phillips-Howard PA, ter Kuile FO, Terlouw DJ, Vulule JM, Ombok M, et al Community-wide effects of permethrin-treated bed nets on child mortality and malaria morbidity in western Kenya. Am J Trop Med Hyg. 2003;68(4 suppl):121–127. 12749495

[15] Clasen TF, Bostoen K, Schmidt W-P, Boisson S, Fung IC-H, Jenkins MW, et al Interventions to improve disposal of human excreta for preventing diarrhoea. Cochrane Libr 2010 Available: http://onlinelibrary.wiley.com/doi/10.1002/14651858.CD007180.pub2/full. Accessed 6 June 2016. 10.1002/14651858.CD007180.pub2 PMC6532559 20556776

[16] Munos MK, Walker CLF, Black RE. The effect of oral rehydration solution and recommended home fluids on diarrhoea mortality. Int J Epidemiol. 2010;39(suppl 1):i75–i87. 10.1093/ije/dyq025 20348131 PMC2845864

[17] Taffa N, Chepngeno G. Determinants of health care seeking for childhood illnesses in Nairobi slums. Trop Med Int Health. 2005;10(3):240–245. 15730508 10.1111/j.1365-3156.2004.01381.x

[18] Ministry of Planning and National Development. Kenya Economic Recovery Strategy for Wealth and Employment Creation 2003–2007. Government of Kenya; 2003 Available: http://siteresources.worldbank.org/KENYAEXTN/Resources/ERS.pdf. Accessed 29 May 2016.

[19] CBS. Kenya Demographic and Health Survey 2003. Calverton, Maryland: CBS, MOH, and ORC Macro; 2004.

[20] KNBS. Kenya Demographic and Health Survey, 2008–09. Calverton, Maryland: KNBS and ICF Macro; 2010.

[21] Ndiritu M, Cowgill KD, Ismail A, Chiphatsi S, Kamau T, Fegan G, et al Immunization coverage and risk factors for failure to immunize within the Expanded Programme on Immunization in Kenya after introduction of new Haemophilus influenzaetype b and hepatitis b virus antigens. BMC Public Health. 2006;6(132). 10.1186/1471-2458-6-132 PMC1475578 16707013

[22] Ministry of Health. National Malaria Strategy 2001–2010. Nairobi: Ministry of Health, Government of Kenya; 2001 [cited 2016 May 13].

[23] Ministry of Public Health and Sanitation. National strategy on infant and young child feeding 2007 to 2010. Nairobi: Ministry of Public Health and Sanitation, Government of Kenya; 2007 Available: https://extranet.who.int/nutrition/gina/sites/default/files/KEN%202007%20National%20Strategy%20on%20Infant%20and%20Young%20Child%20Feeding.pdf. Accessed 13 May 2016.

[24] Wamae A, Kichamu G, Kundu F, Muhunzu I. Child Health Services in Kenya. Kenya Working Papers No. 2. Calverton, Maryland, USA: Macro International Inc.; 2009. Accessed 19 May 2016.

[25] Maina T, Kirigia D. Annual Evaluation of the Abolition of User Fees at Primary Healthcare Facilities in Kenya. Washington DC: Futures Group, Health Policy Project; 2015.

[26] Chuma J, Maina T. Free maternal care and removal of user fees at primary-level facilities in Kenya Monitoring the implementation and impact: Baseline report. Washington, DC: Health Policy Project, Futures Group 2013.

[27] Smith LC, Ruel MT, Ndiaye A. Why Is Child Malnutrition Lower in Urban Than in Rural Areas? Evidence from 36 Developing Countries. World Dev. 2005 8;33(8):1285–305.

[28] Fotso J-C. Urban–rural differentials in child malnutrition: Trends and socioeconomic correlates in sub-Saharan Africa. Health Place. 2007 3;13(1):205–23. 16563851 10.1016/j.healthplace.2006.01.004

[29] Menon P, Ruel MT, Morris SS. Socio-Economic Differentials in Child Stunting are Consistently Larger in Urban than in Rural Areas. Food Nutr Bull. 2000 9 1;21(3):282–9.

[30] Van de Poel E, O’Donnell O, Van Doorslaer E. Are urban children really healthier? Evidence from 47 developing countries. Soc Sci Med. 2007 11;65(10):1986–2003. 17698272 10.1016/j.socscimed.2007.06.032

[31] Matanda DJ, Mittelmark MB, Kigaru DM. Child undernutrition in Kenya: trend analyses from 1993 to 2008–09. BMC Pediatr. 2014;14(1):5. 24410931 10.1186/1471-2431-14-5 PMC3898409

[32] Okeke TA, Okeibunor JC. Rural–urban differences in health-seeking for the treatment of childhood malaria in south-east Nigeria. Health Policy. 2010 4;95(1):62–8. 10.1016/j.healthpol.2009.11.005 20004038

[33] Chuma J, Gilson L, Molyneux C. Treatment-seeking behaviour, cost burdens and coping strategies among rural and urban households in Coastal Kenya: an equity analysis. Trop Med Int Health. 2007 5 1;12(5):673–86. 17445135 10.1111/j.1365-3156.2007.01825.x

[34] Matanda DJ, Mittelmark MB, Kigaru DMD. Breast-, complementary and bottle-feeding practices in Kenya: stagnant trends were experienced from 1998 to 2009. Nutr Res. 2014 6;34(6):507–17. 10.1016/j.nutres.2014.05.004 25026918

[35] Moïsi JC, Gatakaa H, Noor AM, Williams TN, Bauni E, Tsofa B, et al Geographic access to care is not a determinant of child mortality in a rural Kenyan setting with high health facility density. BMC Public Health. 2010;10(1):142. 20236537 10.1186/1471-2458-10-142 PMC2848200

[36] Noor AM, Omumbo JA, Amin AA, Zurovac D, Snow RW. Wealth, mother’s education and physical access as determinants of retail sector net use in rural Kenya. Malar J. 2006;5(1):5. 16436216 10.1186/1475-2875-5-5 PMC1363723

[37] van Eijk AM, Bles HM, Odhiambo F, Ayisi JG, Blokland IE, Rosen DH, et al Use of antenatal services and delivery care among women in rural western Kenya: a community based survey. Reprod Health. 2006;3(1):2. 16597344 10.1186/1742-4755-3-2 PMC1459114

[38] WHO. Indicators for assessing infant and young child feeding practices: Part II Measurement. World Health Organization; 2010. Available: http://www.who.int/nutrition/publications/infantfeeding/9789241599290/en/. Accessed 27 May 2016.

[39] Rutstein SO, Johnson K. The DHS wealth index (DHS Comparative Reports No. 6). Calverton ORC Macro. 2004.

[40] Noor AM, Amin AA, Akhwale WS, Snow RW. Increasing coverage and decreasing inequity in insecticide-treated bed net use among rural Kenyan children. PLoS Med. 2007;4(8):e255 17713981 10.1371/journal.pmed.0040255 PMC1949846

[41] Akumu AO, English M, Scott JAG, Griffiths UK. Economic evaluation of delivering Haemophilus influenzae type b vaccine in routine immunization services in Kenya. Bull World Health Organ. 2007 7;85(7):511–8. 17768499 10.2471/BLT.06.034686 PMC2636374

[42] Vijayaraghavan M, Martin RM, Sangrujee N, Kimani GN, Oyombe S, Kalu A, et al Measles supplemental immunization activities improve measles vaccine coverage and equity: Evidence from Kenya, 2002. Health Policy. 2007;83(1):27–36. 17174435 10.1016/j.healthpol.2006.11.008

[43] Ministry of Public Health and Sanitation. Policy Guidelines on Control and Management of Diarrhoeal Diseases in Children Below Five Years. Nairobi: Ministry of Public Health and Sanitation, Government of Kenya; 2010 Available: https://www.path.org/files/kenya-diarrhoea-policy.pdf. Accessed 13 May 2016.

[44] Blum LS, Oria PA, Olson CK, Breiman RF, Ram PK. Examining the use of oral rehydration salts and other oral rehydration therapy for childhood diarrhea in Kenya. Am J Trop Med Hyg. 2011;85(6):1126–1133. 10.4269/ajtmh.2011.11-0171 22144457 PMC3225165

[45] Olson CK, Blum LS, Patel KN, Oria PA, Feikin DR, Laserson KF, et al Community case management of childhood diarrhea in a setting with declining use of oral rehydration therapy: findings from cross-sectional studies among primary household caregivers, Kenya, 2007. Am J Trop Med Hyg. 2011;85(6):1134–1140. 10.4269/ajtmh.2011.11-0178 22144458 PMC3225166

[46] Arimond M, Ruel MT. Dietary diversity is associated with child nutritional status: evidence from 11 demographic and health surveys. J Nutr. 2004;134(10):2579–2585. 15465751 10.1093/jn/134.10.2579

[47] Bwibo NO, Neumann CG. The need for animal source foods by Kenyan children. J Nutr. 2003;133(11):3936S–3940S. 14672293 10.1093/jn/133.11.3936S

[48] Stephenson K, Amthor R, Mallowa S, Nungo R, Maziya-Dixon B, Gichuki S, et al Consuming cassava as a staple food places children 2–5 years old at risk for inadequate protein intake, an observational study in Kenya and Nigeria. Nutr J. 2010 2 26;9(1):9. 20187960 10.1186/1475-2891-9-9 PMC2837613

[49] Bell A, Jones K. The impossibility of separating age, period and cohort effects. Soc Sci Med. 2013;93:163–165. 10.1016/j.socscimed.2013.04.029 23701919

[50] Government of Kenya. RAPID QUALITATIVE ASSESSMENT Beliefs and attitudes around infant and young child feeding in Kenya. Nairobi: Ministry of Health; 2011.

[51] Magadi MA, Madise NJ, Rodrigues RN. Frequency and timing of antenatal care in Kenya: explaining the variations between women of different communities. Soc Sci Med. 2000;51(4):551–561. 10868670 10.1016/s0277-9536(99)00495-5

[52] Say L, Raine R. A systematic review of inequalities in the use of maternal health care in developing countries: examining the scale of the problem and the importance of context. Bull World Health Organ. 2007 10;85(10):812–9. 18038064 10.2471/BLT.06.035659 PMC2636485

[53] Yesuf EA, Calderon-Margalit R. Disparities in the use of antenatal care service in Ethiopia over a period of fifteen years. BMC Pregnancy Childbirth. 2013;13:131 10.1186/1471-2393-13-131 23767975 PMC3689630

[54] Abuya BA, Onsomu EO, Kimani JK, Moore D. Influence of Maternal Education on Child Immunization and Stunting in Kenya. Matern Child Health J. 2010 9 17;15(8):1389–99. 10.1007/s10995-010-0670-z 20848172

[55] Magadi MA, Agwanda AO, Obare FO. A comparative analysis of the use of maternal health services between teenagers and older mothers in sub-Saharan Africa: Evidence from Demographic and Health Surveys (DHS). Soc Sci Med. 2007 3;64(6):1311–25. 17174017 10.1016/j.socscimed.2006.11.004

[56] Masmalai A, Thongthai V, XiuShi Yang, Richter K. The effect of unwanted pregnancy on prenatal care practice in Thailand. J Popul Soc Stud. 2010;19(1):123–37.

[57] Marston C, Cleland J. Do unintended pregnancies carried to term lead to adverse outcomes for mother and child? An assessment in five developing countries. Popul Stud. 2003 1 1;57(1):77–93. 10.1080/0032472032000061749 12745811

[58] Pagnini DL, Reichman NE. Psychosocial Factors and the Timing of Prenatal Care among Women in New Jersey’s HealthStart Program. Fam Plann Perspect. 2000;32(2):56–64. 10779236

[59] Baume CA, Marin MC. Intra-household Mosquito Net Use in Ethiopia, Ghana, Mali, Nigeria, Senegal, and Zambia: Are Nets Being Used? Who in the Household Uses Them? Am J Trop Med Hyg. 2007 11 1;77(5):963–71. 17984361

[60] Yeager BAC, Huttly SRA, Bartolini R, Rojas M, Lanata CF. Defecation practices of young children in a Peruvian shanty town. Soc Sci Med. 1999 8;49(4):531–41. 10414812 10.1016/s0277-9536(99)00119-7

[61] Boerma JT, Sommerfelt AE. Demographic and health surveys (DHS): contributions and limitations. World Health Stat Q Rapp Trimest Stat Sanit Mond. 1992;46(4):222–226. 8017081

[62] Coughlin SS. Recall bias in epidemiologic studies. J Clin Epidemiol. 1990;43(1):87–91. 2319285 10.1016/0895-4356(90)90060-3

[63] Clarke PM, Fiebig DG, Gerdtham U-G. Optimal recall length in survey design. J Health Econ. 2008;27(5):1275–1284. 10.1016/j.jhealeco.2008.05.012 18667254

[64] Yawn BP, Suman VJ, Jacobsen SJ. Maternal recall of distant pregnancy events. J Clin Epidemiol. 1998;51(5):399–405. 9619967 10.1016/s0895-4356(97)00304-1

[65] Cho H, Salmon CT. Unintended effects of health communication campaigns. J Commun. 2007;57(2):293–317.

[66] Kreps GL. Health Communication Inquiry and Health Promotion: A State of the Art Review. J Nat Sci. 2015 2 1;1(2):35.

